# RNA Interference of Genes Encoding the Vacuolar-ATPase in *Liriomyza trifolii*

**DOI:** 10.3390/insects12010041

**Published:** 2021-01-06

**Authors:** Ya-Wen Chang, Yu-Cheng Wang, Xiao-Xiang Zhang, Junaid Iqbal, Yu-Zhou Du

**Affiliations:** 1 College of Horticulture and Plant Protection & Institute of Applied Entomology, Yangzhou University, Yangzhou 225009, China; dx120170076@yzu.edu.cn (Y.-W.C.); mx120190636@yzu.edu.cn (Y.-C.W.); mx120180613@yzu.edu.cn (X.-X.Z.); junaidagri1@gmail.com (J.I.); 2 Joint International Research Laboratory of Agriculture and Agri-Product Safety, Yangzhou University, Yangzhou 225009, China

**Keywords:** *Liriomyza trifolii*, *V-ATPase*, microinjection, RNAi, mortality

## Abstract

**Simple Summary:**

*Liriomyza trifolii* is an important insect pest of many horticultural and vegetable crops, and it is also an important alien pest, which is increasingly harmful in most parts of China. Here, a dsRNA delivery method was established for *L. trifolii* and used to study two vacuolar-ATPase subunits (*V-ATPase B* and *V-ATPase D*) by microinjection. The dsRNA constructs reduced transcription of both *V-ATPases*, and the knockdown of these genes resulted in increased mortality. This study is the first report on the RNA interference (RNAi)-based approach for *Liriomyza* spp., internal feeders. The use of RNAi technology in *L. trifolii* will contribute to the functional analysis of critical genes and may potentially provide a new idea for control strategies.

**Abstract:**

The leafminer fly, *Liriomyza trifolii*, is an invasive pest of vegetable and horticultural crops in China. In this study, a microinjection method based on dsRNA was developed for RNA interference (RNAi) in *L. trifolii* using genes encoding vacuolar-ATPase (*V-ATPase*). Expression analysis indicated that *V-ATPase B* and *V-ATPase D* were more highly expressed in *L. trifolii* adults than in larvae or pupae. Microinjection experiments with *dsV-ATPase B* and *dsV-ATPase D* were conducted to evaluate the efficacy of RNAi in *L. trifolii* adults. Expression analysis indicated that microinjection with 100 ng *dsV-ATPase B* or *dsV-ATPase* led to a significant reduction in *V-ATPase* transcripts as compared to that of the *dsGFP* control (dsRNA specific to green fluorescent protein). Furthermore, lower dsRNA concentrations were also effective in reducing the expression of target genes when delivered by microinjection. Mortality was significantly higher in *dsV-ATPase B-* and *dsV-ATPase D*-treated insects than in controls injected with *dsGFP*. The successful deployment of RNAi in *L. trifolii* will facilitate functional analyses of vital genes in this economically-important pest and may ultimately result in new control strategies.

## 1. Introduction

*Liriomyza trifolii* (Burgess) (Diptera: Agromyzidae) is an invasive insect pest in both field and greenhouse settings [1]. The larvae of *L. trifolii* injure plants by producing tunnels in leaf tissue, whereas adult females pierce foliage during oviposition [2,3,4]. Although *L. trifolii* was initially reported in the Americas, it has disseminated on a global scale [5]. In China, *L. trifolii* was first reported in Guangdong in 2005, and it is currently a problem in at least 10 provinces [6,7]. *L. trifolii*, *L. sativae,* and *L. huidobrensis*, comprise a group of three leaf miner flies that damage vegetables in China [8,9,10]. Although these three leafminers share similar features, the phenomenon of species displacement has been observed in some regions. For example, shortly after the initial finding of *L. trifolii*, this species displaced *L. sativae* to become the dominant *Liriomyza* spp. in certain parts of southern China [6,7,9,11,12]. *L. trifolii* typically shows greater pesticide resistance than does *L. sativae*, and it can withstand extreme temperatures; furthermore, it has the potential to spread to northern China [13,14,15,16,17].

The most common management strategy for *L. trifolii* control is the use of chemical pesticides, which have been widely used in the field [7,13,14]. Although chemicals have been an effective and convenient option for control of *L. trifolii*, widespread use has caused the emergence of resistant leafminer populations; this change altered interspecific competition with related species and has resulted in serious pesticide pollution [13,18,19,20,21,22]. Therefore, it is imperative to explore alternative control strategies for this pest. The deployment of RNA interference (RNAi) is a highly-specific approach that inhibits gene expression at the post-transcriptional level by fostering the degradation of cognate RNA transcripts [23,24]. RNAi is a useful approach to investigate gene function due to the observable phenotypes that occur when a target gene is silenced [25]. Furthermore, ingested double-stranded RNA (dsRNA) may have insecticidal properties [26,27,28].

A potential candidate for RNAi via injection or ingestion is vacuolar-ATPase (V-ATPase), a highly-conserved enzyme [29,30]. V-ATPase catalyzes the hydrolysis of ATP to ADP and phosphate; the energy from this reaction pumps H+ across membranes and regulates pH in multiple organelles [31]. In a prior study, dsRNA that targeted *V-ATPase A*, which encodes vacuolar ATP synthase subunit A, was lethal to *Diabrotica virgifera* larvae [29]. Furthermore, *dsV-ATPase B* and *dsV-ATPase D* ingestion and injection decreased transcription of target genes in *Peregrinus maidis,* the corn planthopper [30]. In order to establish an RNAi system in *Frankliniella occidentalis*, Badillo-Vargas et al. [32] used a microinjection system to deliver *dsV-ATPase B* directly into the hemocoel of female thrips [32].

In this research, RNAi was used to target mRNAs critical for V-ATPase function. The goal was to develop an RNAi-based approach for *Liriomyza* spp. that can be used to investigate gene function, which will ultimately contribute to better control methods.

## 2. Materials and Methods

### 2.1. Insects

*L. trifolii* was maintained in the laboratory at 25 ± 1 °C with a 16 h light/8 h dark photoperiod [33]. Beans (*Vigna unguiculata*) were used to rear larvae and adults, and foliage with tunnels was collected for pupation.

### 2.2. Cloning, Sequence Alignment, and Expression of V-ATPase Genes

The RNeasy reagent (Vazyme, Nanjing, China) was used to isolate total RNA from *L. trifolii*. RNA purity and integrity were determined by spectrophotometry (Thermo NanoDrop One, Madison, WI, USA) and agarose gel electrophoresis. Based on transcriptome data, we selected *V-ATPase B* and *V-ATPase D* for further study. Partial fragments of the two V-ATPase genes were amplified using specific primers (Table 1), and 5′- and 3′- Rapid amplification of cDNA ends (5’- and 3’-RACE) were used to obtain complete cDNAs as described [17].

Full-length cDNAs of the two V-ATPase genes were queried against other insect *V-ATPases* using BLAST programs (http://www.ncbi.nlm.gov/BLAST/). Sequences were aligned with Clustal X [34], and open reading frames (ORFs) were identified with ORF Finder (https://www.ncbi.nlm.nih.gov/orffinder/). Tools available on the ExPASy Molecular Biology Server (Swiss Institute of Bioinformatics, Switzerland) were deployed to analyze V-ATPase sequences. MEGA 6.0 [35] and the neighbor-joining method were used to create phylogenetic trees of V-ATPases with the following parameters: Poisson correction model, pairwise deletion, and 1000 bootstrap replicates (random seed).

Developmental stages included 3rd instar larvae, two-day-old pupae, and adults (*n* = 10), and treatments contained three independent biological replicates. Total RNA (0.5 μg) was reverse-transcribed using the Bio-Rad iScript™ cDNA Synthesis Kit (Bio-Rad, CA, USA). qRT-PCR was executed with gene-specific primers (Table 1) in 20 μL volumes as described [36]. Reactions were conducted with a CFX-96 real-time PCR system (Bio-Rad Laboratories, Berkeley, CA, USA). Each treatment contained four replicates, and each reaction was performed in triplicate.

### 2.3. Synthesis of dsRNA and Delivery by Microinjection

Two full-length *L. trifolii* V-ATPase genes were identified using the online website (http://sidirect2.rnai.jp/); the regions for RNA silencing were determined, and primers for RNAi were designed. Sense and antisense primers included a T7 promoter sequence (TAATACGACTCACTATAGGGAGA) at the 5′ ends to catalyze transcription from both cDNA strands (Table 1). dsRNA specific to the gene encoding green florescence protein (*GFP*) was used as a control (Table 1). PCR products were inserted into pGEM-T easy (Promega, Madison, WI, USA), and resulting constructs were used as template DNA in subsequent amplifications. Purified DNA templates (1.5 µg) were used for in vitro dsRNA synthesis and purified using the MEGAscript™ RNAi Kit (Thermo, Waltham, MA, USA, #AM1626) as recommended by the company. The quality of dsRNA was evaluated by spectrophotometry and gel electrophoresis.

**Figure 1:**
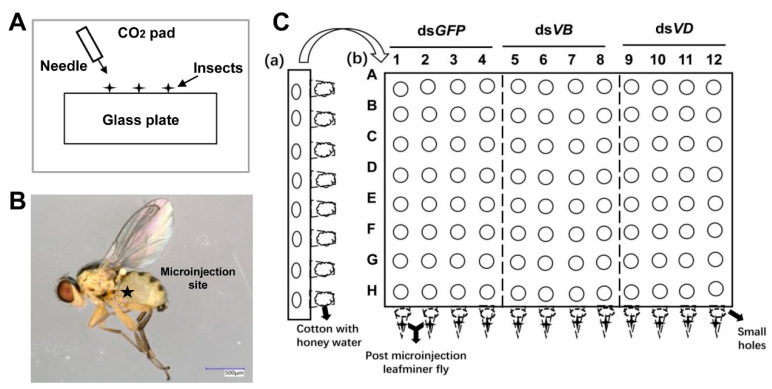
Schematic diagram of the microinjection method (**A**), injection site (**B**), and post-injection feeding device (**C**) used in this study. dsGFP: dsRNA of green fluorescent protein gene, dsVB: dsRNA of vacuolar-ATPase B gene, dsVD: dsRNA of vacuolar-ATPase D gene.

After anesthesia with CO_2_, newly-emerged *L. trifolii* adults were microinjected (WPI nanoliter 2010, Sarasota, FL, USA) with *dsGFP* (5 nL) or an equal volume of *dsV-ATPases* at different concentrations. Injected insects were then caged on modified 96-wells plate supplied with a honey-water solution and monitored daily until the experiments ended (Figure 1).

### 2.4. Analysis of Silencing Efficiency

Time course and concentration experiments were performed to evaluate the impact of *dsRNA* injection on newly-emerged adults using the experimental design described above. The experiment was performed with three replicates per treatment, and *dsGFP* was included as a control. In time course experiments, approximately 100 newly-emerged adults were injected with 5 nL (100 ng) of dsRNA for each gene (*V-ATPase B*, *V-ATPase D,* or *GFP*). Injected adults in each treatment (*n* = 100) were divided into three, modified 96-well plates representing 12, 24, and 48 h post-microinjection, and each treatment of about 30 newly-emerged adults constituted one biological repetition. Two concentrations (25 and 50 ng) were selected for further analysis, and silencing efficiency was detected 24 h post-injection. The number of adults in each treatment was the same as time course experiments. Live adults were collected for RNA extraction, and silencing efficiency was analyzed by qRT-PCR. In addition, survival rates were calculated for each *dsRNA* group (100 ng) and contained 60 micro-injected adults (10 individuals represented one repetition); the numbers of dead and live adults were recorded every 12 h for 5 days (120 h) post-injection.

### 2.5. Statistical Analyses

The quantity of V-ATPase genes at different developmental stages was calculated using the 2^−ΔΔCt^ method; *ACTIN* served as an internal reference gene [36]. One-way ANOVA was implemented to identify significant differences in mRNA levels, followed by Tukey’s multiple comparison (*p* < 0.05) and analysis with SPSS v. 16.0 (SPSS, Chicago, IL, USA). For ANOVA, data were transformed for the homogeneity of variances test.

For silencing efficiency, the relative abundance of target genes (*V-ATPase B* or *V-ATPase D*) and survival rates were compared to the control (*dsGFP*). The normalized values for treatment samples were divided by mean normalized values for the control. The Student’s t-test was performed to compare different treatments (*p* < 0.05) using SPSS v. 16.0.

## 3. Results

### 3.1. Sequence Analysis and Expression Patterns of V-ATPase Genes in L. trifolii

**Figure 2:**
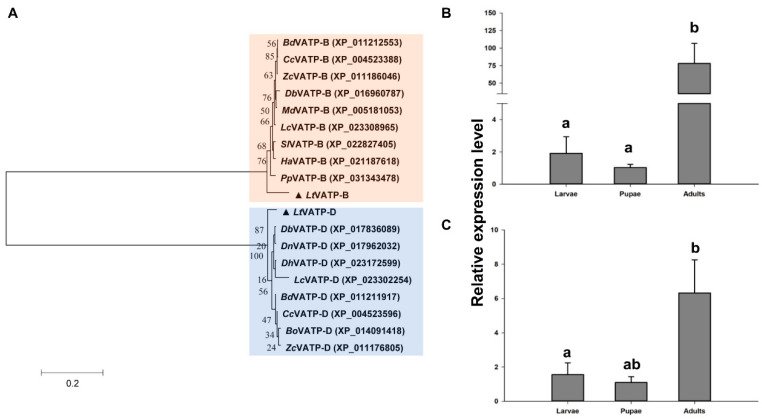
Neighbor-joining phylogenetic tree and relative expression analysis of *V-ATPase B* and *V-ATPase D*. (**A**) Phylogenetic tree. *L. trifolii* V-ATPase B and D are labeled with triangles. Numbers on the branches represent bootstrap values obtained from 1000 replicates (only bootstrap values > 50 are shown). Species names and accession numbers are listed in Table S1. (**B**) Relative expression of *V-ATPase B* and (**C**) *V-ATPase D* in *L. trifolii* larvae, pupae, and adults. Different lowercase letters in panels (**B**,**C**) indicate significant differences between developmental stages. Tukey’s multiple range test was used for pairwise comparison of means (*p* < 0.05).

Two cDNA sequences encoding V-ATPase subunits, *V-ATPase B* and *V-ATPase D,* were identified. Full-length cDNA sequences of *V-ATPase B* (1974 bp) and *V-ATPase D* (925 bp) were obtained from RACE and submitted to GenBank as accession nos. MT776906 and MT776907, respectively. The predicted ORFs for *V-ATPase B* and *V-ATPase D* were 1617 bp and 744 bp, respectively. Basic local alignment search tool (BLAST) analysis of predicted amino acid sequences showed that V-ATPase B and D subunits are conserved and share over 90% sequence identity with other members of Diptera (Figure S1). A phylogenetic tree was obtained using the amino acid sequences of 17 V-ATPases in Diptera, including nine V-ATPase B and eight V-ATPase D sequences. The phylogenetic tree contained two distinct clusters containing V-ATPase B and V-ATPase D (Figure 2A). Specifically, V-ATPase B showed 76% phylogenetic identity with selected other Diptera V-ATPase B subunits and V-ATPase D showed 87% phylogenetic identity with selected Diptera V-ATPase D subunits.

The expression levels of *V-ATPase B* and *V-ATPase D* were monitored in 3rd instar larvae, two-day-old pupae, and *L. trifolii* adults. Both *V-ATPase B* and *V-ATPase D* exhibited significant differences in expression levels during development (*V-ATPase B*: *F*_2,6_ = 28.926, *p <* 0.05; *V-ATPase D*: *F*_2,6_ = 6.304, *p <* 0.05). Expression levels of *V-ATPase B* and *V-ATPase D* were highest in *L. trifolii* adults and were 78.12- and 6.33-fold higher than expression in pupae. There were no significant differences in *V-ATPase* expression between larval and pupal forms of *L. trifolii* (Figure 2B,C).

### 3.2. Knockdown of V-ATPase B and V-ATPase D Expression

**Figure 3:**
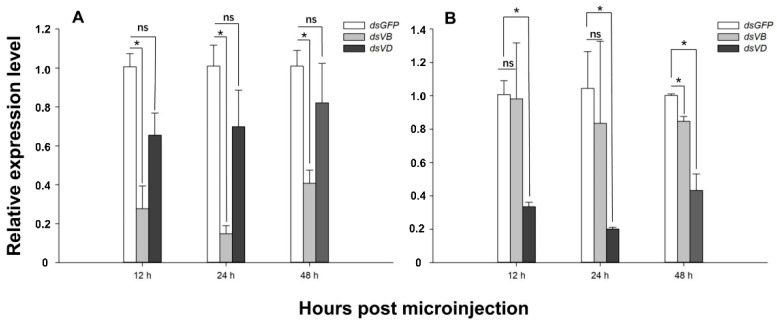
Abundance of *V-ATPase B* and *V-ATPase D* transcripts in *L. trifolii* after microinjection with dsRNA. Adults were injected with 100 ng *dsV-ATPase B*, *dsV-ATPase D*, or *dsGFP* (control), and expression was measured at 12, 24, and 48 h. Panels: (**A**) *V-ATPase B* and (**B**) *V-ATPase D* expression. Data were analyzed by the Student’s *t*-test, *p* < 0.05. Asterisks represent significant differences between control and RNAi treatment; ns indicates no significant differences.

RNA interference studies were conducted by microinjection of *L. trifolii* adults with dsRNA of *V-ATPase B* and *V-ATPase D*. There was a significant (>60%) reduction in *V-ATPase B* expression when newly-emerged adults were injected with *dsV-ATPase B* as compared to that with *dsGFP* at different times post-injection (12 h: *t* = 5.437, *p <* 0.05; 24 h: *t* = 7.485, *p <* 0.05; 48 h: *t* = 5.729, *p <* 0.05) (Figure 3A). *V-ATPase B* expression was not significantly different in samples injected with *dsV-ATPase D* (12 h: *t* = 2.679, *p =* 0.055; 24 h: *t* = 1.437, *p =* 0.224; 48 h: *t* = 0.864, *p =* 0.460). Similar expression patterns were observed in RNAi experiments with *V-ATPase D* (*V-ATPase D*—*dsV-ATPase D*, 12 h: *t* = 7.717, *p <* 0.05; 24 h: *t* = 3.800, *p <* 0.05; 48 h: *t* = 5.779, *p <* 0.05; *V-ATPase D*—*dsV-ATPase B*, 12 h: *t* = 0.072, *p =* 0.949; 24 h: *t* = 0.388, *p =* 0.718). However, the expression of *V-ATPase D* in samples injected with *dsV-ATPase B* at 48 h post-injection was significantly different from that of the *dsGFP* control (48 h: *t* = 4.983, *p <* 0.05) (Figure 3B). When *L. trifolii* was injected with 100 ng *dsV-ATPase D*, silencing efficiency was highest at 24 h post-injection, and interference efficiency began to decrease at 48 h (Figure 3).

**Figure 4:**
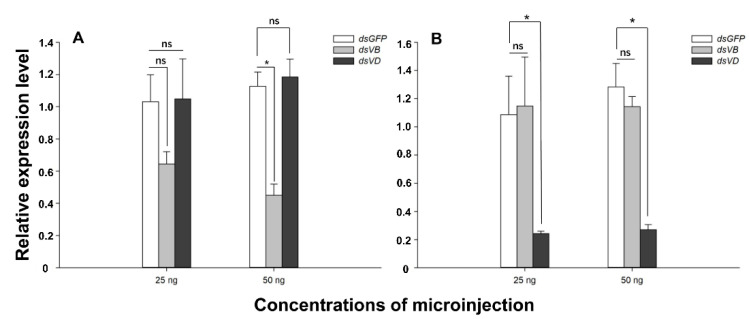
Expression of *V-ATPase B* and *V-ATPase D* in *L. trifolii* injected with different dsRNA concentrations. Adults were injected with 25 or 50 ng *dsV-ATPase B*, *dsV-ATPase D*, or *dsGFP* (control) and expression was measured at 24 h. Panels: (**A**) *V-ATPase B* and (**B**) *V-ATPase D* expression. Data were analyzed with the Student’s *t*-test, *p* < 0.05. Asterisks represent significant differences; ns indicates no significant difference.

There was no significant difference in *V-ATPase B* expression in *L. trifolii* injected with 25 ng *dsV-ATPase B*, compared with that of the *dsGFP* injection group (25 ng: *t* = 2.120, *p =* 0.101) (Figure 4A). However, *V-ATPase B* expression was significantly lower when 50 ng of *V-ATPase B* was microinjected as compared to expression when 50 ng *dsGFP* was injected (50 ng: *t* = 6.062, *p <* 0.05) (Figure 4A). Injection of both 25 ng and 50 ng *dsV-ATPase D* caused significant reduction in *V-ATPase D* expression as compared to 25 or 50 ng *dsGFP* (25 ng: *t* = 3.094, *p <* 0.05; 50 ng: *t* = 5.921, *p <* 0.05) (Figure 4B). No cross-interference activity was identified between the two *dsV-ATPases* (*V-ATPase B*—*dsV-ATPase D*, 25 ng: *t* = −0.056, *p =* 0.958; 50 ng: *t* = −0.410, *p =* 0.704; *V-ATPase D*—*dsV-ATPase B*, 25 ng: *t* = −0.136, *p =* 0.899; 50 ng: *t* = 0.363, *p =* 0.482).

### 3.3. Mortality of L. trifolii Injected with dsV-ATPase-B and dsV-ATPase-D

**Figure 5:**
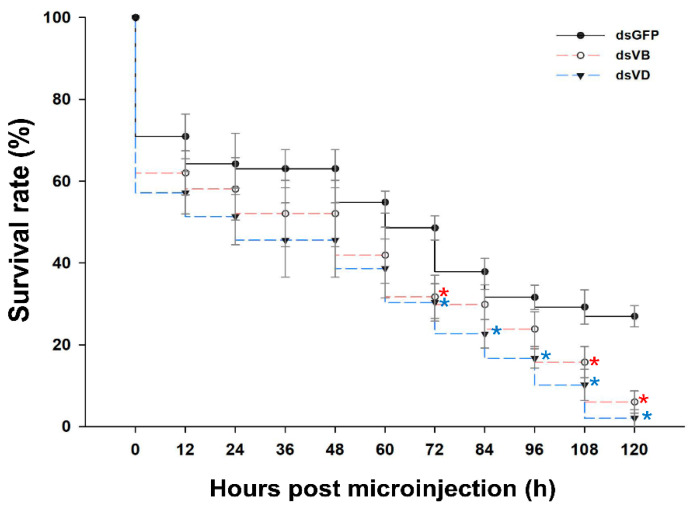
*L. trifolii* mortality after ingestion of dsRNA. Adults were injected with 100 ng *dsV-ATPase B*, *dsV-ATPase D*, or *dsGFP* (control) and mortality was measured at 12 h intervals. Asterisks represent significant differences between controls and RNAi treatment.

*L. trifolii* adults were injected with *dsV-ATPase B, dsV-ATPase D,* or *dsGFP* (control), and mortality was assessed at 12 h intervals for five days. In general, adults injected with *dsV-ATPase B* and *dsV-ATPase D* had similar survival rates throughout the experiment (120 h) (Figure 5). Significant reductions in survival relative to that of the control were first observed for *dsV-ATPase B* and *dsV-ATPase D* at 72 h after injection (*V-ATPase B*: *t* = 2.806, *p <* 0.05; *V-ATPase D*: *t* = 3.364, *p <* 0.05). Survival in the *dsV-ATPase D* treatment group continued to decline significantly at 84, 96, 108, and 120 h after injection (84 h: *t* = 3.180, *p <* 0.05; 96 h: *t* = 3.934, *p <* 0.05; 108 h: *t* = 3.362, *p <* 0.05; 120 h: *t* = 7.534, *p <* 0.05). In addition to the 72 h interval, survival in the *dsV-ATPase B* treatment group was also significantly lower at 108 and 120 h after injection (108 h: *t* = 2.382, *p <* 0.05; 120 h: *t* = 5.630, *p <* 0.05). At five days (120 h) after injection, only 6.02% and 2.08% of adults survived injection with *dsV-ATPase B* and *V-ATPase D*, respectively, compared to 26.98% with *dsGFP* (Figure 5).

## 4. Discussion

In this study, two V-ATPase subunits, V-ATPase B and D, were identified in *L. trifolii*. A phylogenetic tree was created providing further support to the correct identity of both V-ATPase gene sequences [32], which were then selected as target genes for our RNAi studies. The expression of target genes at different developmental stages is a critical component of successfully using RNAi. In this study, the relative expression levels of *V-ATPases* in *L. trifolii* adults were significantly higher in larvae than pupae. RNAi is frequently utilized to explore insect gene function; however, it is important to note that the optimal stage for interference and dsRNA concentrations are gene- and species-specific [37,38]. For example, studies have determined that *Periplaneta americana*, *Locusta migratoria*, *Zophobas atratus*, and *Spodoptera litura* show varying sensitivities to RNAi [39].

*V-ATPase* subunits are essential genes and effective targets for RNAi [29,30,40]. The membrane protein V-ATPase catalyzes ATP hydrolysis; this results in the expulsion of H+ across membranes and against a concentration gradient [30,41]. Multiple *V-ATPase* subunits have been selected as RNAi targets in insects [32]; for example, the knockdown of *V-ATPase B* in *Drosophila melanogaster* resulted in 100% mortality [42]. Baum et al. [29] used artificial diets amended with *dsV-ATPase A* or *dsV-ATPase D* dsRNAs and reported negative effects on growth and survival of several Coleopteran species. When *V-ATPase E* was silenced, increased mortality was observed for *Manduca sexta, D. melanogaster*, *Tribolium castaneum*, and *Acyrthosiphon pisum* [43]. When *Nilaparvata lugens* was fed with three different forms of *dsV-ATPase E*, transcription was reduced by 41%, 55%, and 48% [40]. In the Colorado potato beetle, *V-ATPase B* and *V-ATPase E* transcripts were reduced by 81% and 59%, respectively, when corresponding dsRNAs were encapsulated in heat-killed bacteria and applied to potato leaves [44]. Wuriyanghan et al. [45] demonstrated silencing of a *V-ATPase* gene in the psyllid *Bactericerca cockerelli* via oral delivery of dsRNA or small interfering RNA. In *P. maidis*, the silencing of *V-ATPase B* and *V-ATPase D* reduced fecundity and increased mortality when dsRNAs were ingested or injected into the hemocoel [30].

In this study, we used a microinjection method to introduce *dsV-ATPases* into *L. trifolii* and showed that the relative expression levels of *V-ATPase B* and *V-ATPase D* decreased at 12, 24, and 48 h after injection. At 48 h post-injection, RNAi efficiency decreased in adults injected with 100 ng *dsV-ATPase B* or *dsV-ATPase D*, suggesting that catabolism of dsRNA molecules was underway. With the exception of the 48 h time point, *dsV-ATPase B* was significantly reduced after *dsV-ATPase D* injection, suggesting an interaction between *V-ATPase B* and *V-ATPase D*. The optimal concentration for silencing was determined for both *V-ATPase B* and *V-ATPase D* in *L. trifolii*. It is important to note that surpassing the optimal concentration does not necessarily result in greater silencing [37,38]. The interference efficiency of 100 ng at different post-injection times was significant; therefore, it was necessary to determine interference efficiency at the lower concentrations of 25 and 50 ng. With the exception of 25 ng *dsV-ATPase B*, concentrations of *dsV-ATPases* genes had similar, significant effects (Figure 4).

Although the microinjection of dsRNA into the insect body resulted in a high level of suppression, there are limitations to this delivery system. Side-effects of microinjection include the pressure forced on the insect during the injection process and decreased survival due to the wound at the injection site [24,30,32,46,47]. *Liriomyza* spp. larvae are leafminers [2,3,4], and it is quite difficult to inject eggs and larvae when they reside in leaf tissue. Although *Liriomyza* exit leaf tissue for pupation, it is not trivial to inject pupae under high pressure. Therefore, microinjection of adults was the optimal choice for *L. trifolii.*

In this study, significant mortality was observed in *L. trifolii* 72 h after injection with *dsV-ATPase B* and *dsV-ATPase D*. In *F. occidentalis* [32], significant mortality was observed at six days post-injection. In the western corn rootworm, 100% mortality was observed at 14 days following the knockdown of *V-ATPase* [48]. In bed bugs, gene knockdown and reduced fecundity and survival were observed within one week, but mortality required a longer period of time [49,50]. Mortality does not seem to be a suitable indicator for leafminer flies because relatively long post-injection periods and higher concentrations are required for suppression. Although significant differences were observed, the relatively high mortality rate in dsGFP-injected controls reflects the difficulty of injecting small insects and the subsequent wounding effects. Furthermore, the average life span for *Liriomyza* adults is 1–2 weeks [51], which makes long observation periods impractical. Although microinjection of dsRNA was successful in interfering with the V-ATPase target genes, it is still necessary to explore new methods and indicators for RNAi efficiency in *Liriomyza* adults.

## 5. Conclusions

RNAi is a valuable approach for deciphering gene function in insects. In this investigation, a dsRNA delivery method was established for *L. trifolii* and used to study two V-ATPase subunits. The dsRNA constructs reduced transcription of *V-ATPase B* and *V-ATPase D*, and the knockdown of these genes resulted in increased mortality. The use of RNAi technology in *L. trifolii* will facilitate functional analysis of critical genes and may potentially result in novel control strategies.

## Figures and Tables

**Table 1:** Primers used in cDNA cloning, dsRNA synthesis, and real-time quantitative PCR.

Gene	Primer Sequences (5′→3′)		Fragment Length (bp)
**Primers for cDNA Cloning and Full-Length cDNA Amplification**			
*V-ATPase B*	F	ACAAAACAGTGACAGGCGTGAAT	669
	R	TTGTAGGGTCATTAGCCAGGTTT	
	5′	GCCAGCACTTGTTCGTAGTAGCCTTT	200
	3′	GGTCGTCGTGGTTTCCCTGGTTACAT	920
*V-ATPase D*	F	TCTGCCCGTGTTTGAGTCCTATC	365
	R	CCTGTTGGAGCAGTTCAGCGTTA	
	5′	ACACGGGCAGAGTGACACCAGCAAC	398
	3′	ACGCTGAACTGCTCCAACAGGGTA	196
Primers for dsRNA synthesis			
*dsV-ATPB*	F	TAATACGACTCACTATAGGGAG AACTTTCAATGGGTCTGGTA	412
	R	TAATACGACTCACTATAGGGAG GTCATTAGCCAGGTTTAGAA	
*dsV-ATPD*	F	TAATACGACTCACTATAGGGAG TGAGTCCTATCAAGATGGTT	284
	R	TAATACGACTCACTATAGGGAG TCTTCAAACGATAGAACTCC	
*dsGFP*	F	TAATACGACTCACTATAGGGAGA CCTCGTGACCACCCTGACCTAC	314
	R	TAATACGACTCACTATAGGGAGA CACCTTGATGCCGTTCTTCTGC	
Primers for qRT-PCR			
*V-ATPase B*	F	ACAAAACAGTGACAGGCGTGAATG	92
	R	CGGCAAAGTCAAGTAAACTATCTC	
*V-ATPase D*	F	GGCGGAAGCCAAGTTCACTAC	115
	R	GGGCAGAGTGACACCAGCAAC	
*ACTIN*	F	TTGTATTGGACTCTGGTGACGG	73
	R	GATAGCGTGAGGCAAAGCATAA	

Note: F, forward; R, reverse; 5′, 5′ RACE primer; 3′, 3′ RACE primer; underscored nucleotides represent the T7 polymerase promoter sequence.

## Data Availability

Data is contained within the article or Supplementary Material.

## Supplementary Materials

The following are available online at https://www.mdpi.com/2075-4450/12/1/41/s1, Figure S1: Multiple sequence alignments of V-ATPase B and V-ATPase D deduced amino acid sequences. Letters shaded in black or gray indicate sequence differences. Panes: (A) V-ATPase B; and (B) V-ATPase D, Table S1: Inferred amino acid sequence identities of V-ATPase B and D from *Liriomyza trifolii* with its homologs from other insects.
