# Supplementary material for: RNA Interference of Genes Encoding the Vacuolar-ATPase in Liriomyza trifolii
Source: Insects. 2021 Jan 6;12(1):41. doi: 10.3390/insects12010041 (PMC7825530; doi:10.3390/insects12010041)
Supplement: Supplementary file 1 [file insects-12-00041-s001.zip › Supplementary materials/Table S1.docx]

**Table S1.** Inferred amino acid sequence identities of V-ATPase B and D from *Liriomyza trifolii* with its homologs from other insects.

| Genes | | Species | Accession numbers | | |
| --- | --- | --- | --- | --- | --- |
| *V-ATPase B* | *Spodoptera litura* | | | XP022827405 |  |
|  | *Lucilia cuprina* | | | XP023308965 |  |
|  | *Zeugodacus cucurbitae* | | | XP011186046 |  |
|  | *Bactrocera dorsalis*  *Drosophila biarmipes*  *Ceratitis capitata* | | | XP011212553 |  |
|  |  |  |  | XP016960787 |  |
|  |  |  |  | XP004523388 |  |
|  | *Helicoverpa armigera*  *Photinus pyralis*  *Musca domestica* | | | XP021187618 |  |
|  |  |  |  | XP031343478 |  |
|  |  |  |  | XP005181053 |  |
| *V-ATPase D* | *Zeugodacus cucurbitae*  *Bactrocera dorsalis* | | | XP011176805 |  |
|  |  |  |  | XP011211917 |  |
|  | *Ceratitis capitata* | | | XP004523596 |  |
|  | *Bactrocera oleae* | | | XP014091418 |  |
|  | *Drosophila busckii* | | | XP017836089 |  |
|  | *Drosophila navojoa* | | | XP017962023 |  |
|  | *Drosophila hydei* | | | XP023172599 |  |
|  | *Lucilia cuprina* | | | XP023302254 |  |
