# Supplementary figures and images for: RNA Interference of Genes Encoding the Vacuolar-ATPase in Liriomyza trifolii
Source: Insects. 2021 Jan 6;12(1):41. doi: 10.3390/insects12010041 (PMC7825530; doi:10.3390/insects12010041)

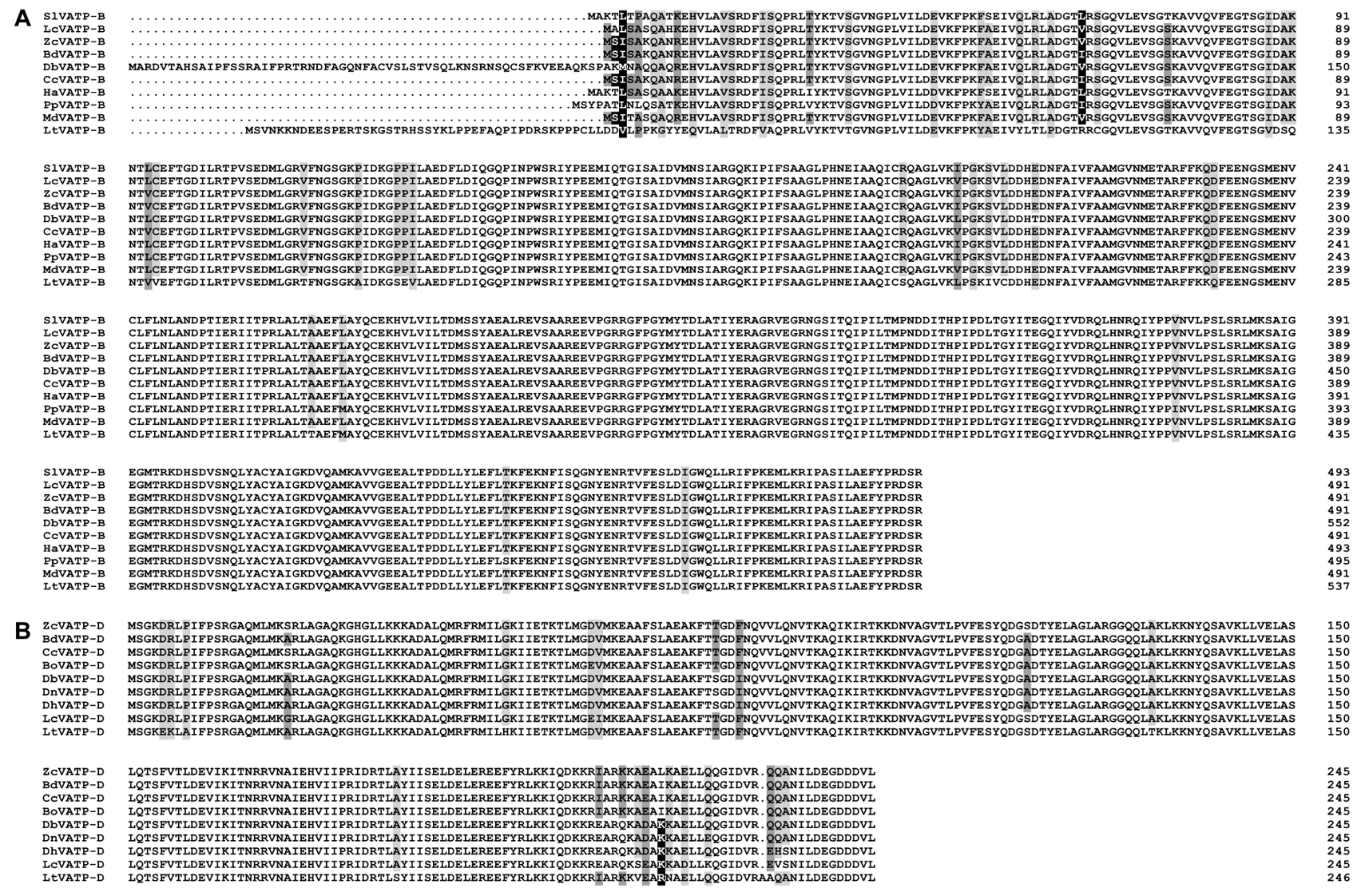

Supplement: Supplementary file 1 [file insects-12-00041-s001.zip › Supplementary materials/Figure S1.tif]
